# Executive Control in Depressive Rumination: Backward Inhibition and Non-inhibitory Switching Performance in a Modified Mixed Antisaccade Task

**DOI:** 10.3389/fpsyg.2017.00136

**Published:** 2017-02-14

**Authors:** Barbara C. Y. Lo, Jeffrey C. C. Liu

**Affiliations:** ^1^Department of Psychology, The University of Hong KongHong Kong, Hong Kong; ^2^Department of Clinical Psychology, Tai Po HospitalHong Kong, Hong Kong

**Keywords:** attention switching, depressive rumination, executive dysfunction, mixed antisaccade task, eye-tracking

## Abstract

Background and Objectives: The present study examines backward inhibition (BI) and non-inhibitory switching performance among depressed and healthy participants in a modified mixed antisaccade task. Specifically, sad and neutral faces were incorporated in the design to examine executive control difficulties associated with brooding trait. Methods: Thirty-nine participants took part in the study, including 19 depressed patients and 20 healthy control subjects. Participants completed a diagnostic interview and self-report questionnaires, including the Beck Depression Inventory and Ruminative Response Scale-Brooding Subscale. They were then instructed to complete prosaccade and antisaccade trials in the pure and mixed blocks whereby eye gazes were tracked to assess inhibition and switching efficiency. Results: For the switching effects, a significant group × brooding × task type interaction was found as hypothesized when multilevel modeling analysis was employed. Switching deficits associated with brooding was found to be greatest when sad faces were presented to depressed group. No significant results in BI or error rates were observed. Conclusion: The patterns observed suggest that as opposed to BI, set shifting difficulty associated with brooding trait may be modulated by negative mood and cognition. In future research, emotional faces other than sad faces may be used to further explore if the observations could be generalized to other affective conditions.

## Introduction

The stability of ruminative tendency across time has been widely demonstrated in longitudinal and field studies (e.g., Just and Alloy, 1997; Bagby et al., 2004), suggesting the possibility that rumination may arise from executive control dysfunctions (Koster et al., 2011; Whitmer and Gotlib, 2013). During a typical ruminative response, intrusive thoughts relating to higher order goals in life (e.g., the failure to secure achievements, relationship problems) are triggered and sustained in such ways that the individual finds it difficult to disengage and shift his or her attention to something else. This specific perseverative nature of rumination has prompted scientists to study it in the context of executive control pertaining to deployment of attention and working memory resources (Koster et al., 2011). One suggestion is that the recurrence of ruminative thoughts may be attributed by poor inhibition abilities (Joormann, 2006). This “inhibition deficit” account has been widely demonstrated in various experimental designs involving working memory updating, including the Stroop task (Krompinger and Simons, 2011), prose distraction task (Lau et al., 2007), memory refresh task (Bernblum and Mor, 2010), negative priming task (Joormann, 2006), and mental sorting task (Joormann et al., 2011). Ruminators seem to not only have weaker ability to ignore irrelevant/no-longer-relevant affective information when updating new information, but also exhibit significant difficulty removing such information from working memory once activated (Joormann and Gotlib, 2008; Berman et al., 2011; Foland-Ross et al., 2013). A second suggestion concerns that the rigidity of rumination processes may arise from set shifting difficulties (Koster et al., 2011). Early studies have highlighted that ruminators exhibit shifting difficulties with neuropsychological tests such as the Wisconsin Card Sorting Test (Davis and Nolen-Hoeksema, 2000). In cognitive tasks, that employ online temporal measures, event-related potential analyses of a modified mental counting task revealed that high ruminators tend to require more neuronal resources when switching between affective information under a sad mood state (Lo et al., 2012). Replications of this task with facial expressions as stimuli also indicated that rumination tendency is associated with greater behavioral switch costs under emotional conditions (De Lissnyder et al., 2012; Koster et al., 2013).

Some researchers hence attempted to examine both inhibitory control and switching performances associated with depressive rumination using a single task-switching paradigm (Whitmer and Banich, 2007; De Lissnyder et al., 2010; Whitmer and Gotlib, 2012). In these paradigms, participants respond to targets based on various operational rules (e.g., comparing the size or orientation of visual stimuli) across trials. Trials in which the operational rule presented differs from that of previous consecutive trials are called switch trials (i.e., A-B sequence), and the reaction times of response presses in these switch trials are compared to those of repeat trials (i.e., A-A sequence) to index switching efficiency. It is assumed that when the individual switch to a new operational rule (i.e., switch to B rule in A-B sequence), the previous rule (i.e., A rule in A-B sequence) has to be inhibited. In the condition that individual has to switch back to the previously abandoned rule in a subsequent trial (i.e., back to A rule in the A-B-A sequence), he/she may take longer to respond than normal switch conditions due to the need to reactivate a previously inhibited mental rule. The difference in reaction times across these inhibition switch and normal switch conditions is referred to as “backward inhibition” (BI) cost to index inhibition control efficiency. It is important to note that in the given example of A-B-A condition, both BI and switching operations are involved when processing the “back to A rule” in the last trial. Hence to deduce a purer index of switching effect, often researchers would compare reaction times of switch trials that do not involve inhibition operations (i.e., A-A-B conditions) with repeat trials (B-B-B conditions) to indicate non-inhibitory switch (NIS) cost (Whitmer and Banich, 2007). In neutral versions of this paradigm, it appears that trait rumination is related to BI deficits but not to NIS difficulties (Whitmer and Banich, 2007; Whitmer and Gotlib, 2012). Yet, when affective content is incorporated, valence-specific inhibition difficulty, as well as general impairment in set shifting, were both observed to be associated with trait rumination (De Lissnyder et al., 2010).

To follow up on this line of work, De Lissnyder et al. (2011) applied the same task-switching paradigm in a mixed antisaccade task to examine rumination associated switching deficits through the detection of eye gaze responses. In particular, the authors argued that measuring direct physiological responses helps minimize response artifacts related to depression-induced motor delays. In prosaccade trials, participants are instructed to look at a target stimulus, whereas in antisaccade trials, participants limit their eye movements and look in the opposite direction when a peripheral visual stimulus is presented. Performance in a mixed block of prosaccade and antisaccade trials is then compared with that of a single block to determine switching efficiency. In contrast, inhibition control deficits were measured by comparing performance between prosaccade and antisaccade trials. According to the authors, no differences in switching performance were found between high and low ruminators in their university sample but high ruminators exhibited significantly slower antisaccade latencies as compared to low ruminators, implying impairments in inhibitory control functions (De Lissnyder et al., 2011). However, their study was limited in that only neutral stimuli (i.e., geometric figures) were tested, and it remains unclear how emotionally charged stimuli presented to depressed patients may affect performance.

In brief, trait rumination appears to be closely associated with inhibition difficulties under general conditions (Whitmer and Banich, 2007; De Lissnyder et al., 2011; Whitmer and Gotlib, 2012), and with switching impairments when emotional material was incorporated (De Lissnyder et al., 2010) based on existing literature. To further explore BI and switching functions associated with depressive rumination in a same task design amongst clinical sample, the present project attempts to replicate De Lissnyder et al.’s (2011) mixed antisaccade design (thereby minimizing response artifacts related to depression-induced motor delays), but with emotional pictures (sad versus neutral faces) incorporated. We expect processing of emotional faces to be particularly impactful because these special images contain interpersonal cues and social meanings, which may further trigger self-referential judgments.

In past literature, Derakshan et al. (2009) was among the first to adopt emotional faces as stimuli in an antisaccade task and examined inhibitory control functions associated with dysphoric mood. The authors showed that dysphoric individuals showed greater difficulty making antisaccades when processing emotional faces. García-Blanco et al. (2013) also examined inhibitory control functions of clinical subjects by adopting emotional faces in an antisaccade task. They recruited a sample of bipolar disorder patients who were in either euthymic, manic or depressive states. Results supported a mood congruency effect that patients with bipolar depression exhibit more difficulties making antisaccades in processing sad faces and patients in manic phase display more problems making antisaccades in processing happy faces. Apart from antisaccade tasks, neuroimaging studies have also adopted the use of emotional facial stimuli to examine the executive control functions associated with depressive states. Adopting a task-switching design, latest fMRI research by Piguet et al. (2016) investigated mood disorder patients’ switching inflexibility and BI abilities as compared to controls in processing sad and happy faces. The patient group showed greater switching difficulty in processing these emotional faces in general, represented by greater behavioral switch costs, and the magnitude of it appeared to be associated with increased activation in fronto-parietal attention networks, suggesting that more neural resources were recruited and patients required greater effort shifting to new mental representations (Piguet et al., 2016). However, no group difference was observed in terms of BI cost.

In all, given that emotional faces are such powerful tool in eliciting affective interference, we aim to adopt an affective version of De Lissnyder et al.’s (2011) mixed antisaccade task in which neutral and sad faces were incorporated. Specifically, maladaptive forms of rumination, namely brooding tendency, would be assessed, and both clinically depressed and healthy control samples recruited to examine how depressive status may modulate the relationship between brooding and affective switching inflexibility, as well as between brooding and BI dysfunctions. Based on existing literature, it is hypothesized that switching deficits associated with brooding would be greatest under emotional conditions (i.e., when sad faces are presented to depressed group), whereas BI deficits associated with brooding would be observed irrespective of mood state and affective experiences.

## Materials and Methods

### Participants

Our sample consisted of 39 participants, including 19 depressed patients (three males and 16 females) and 20 healthy controls (four males and 16 females). Depressed patients were recruited from outpatient psychiatric clinics and had sought clinical psychology services in local hospitals. These individuals were selected based on the following criteria: (i) those older than 18 years of age, (ii) those who suffered from a major depressive disorder based on a structured diagnostic interview – The Structured Clinical Interview for DSM-IV-TR Axis I Disorders (SCID: First et al., 2002), and (iii) those not suffering from psychotic or substance abuse disorders at the time of testing. The healthy controls were adult participants recruited from the local community through personal ties, advertising in churches and volunteer agencies. The depressed group did not differ from the healthy control group in terms of age [*F*(1,38) = 0.473, *p* > 0.05, depressed mean = 41 years old, control mean = 38 years old], years of education [*F*(1,38) = 0.063, *p* > 0.05, depressed mean = 13.45 years, control mean = 13.21 years] or gender distribution (χ^2^ < 1, *p* > 0.05). Not surprisingly, the depressed group showed significantly higher BDI scores [*F*(1,38) = 19.45, *p* < 0.001, depressed mean = 22.25, control mean = 8.15] and RRS-brooding scores [*F*(1,38) = 10.54, *p* < 0.01, depressed mean = 11.85, control mean = 8.85] compared to the healthy controls.

### Materials

#### Diagnostic Interview and Self-report Questionnaires

The Structured Clinical Interview for DSM-IV-TR Axis I Disorders (SCID: First et al., 2002) was administered to screen for depression. The Ruminative Response Scale – brooding subscale (RRS: Nolen-Hoeksema and Morrow, 1991) and Beck Depression Inventory (BDI-II: Beck et al., 1996) were administered to assess participants’ ruminative tendency and depressive severity.

#### Facial Expression Images

Twelve photos each of sad and neutral facial expressions were extracted from the Taiwanese Facial Expression Image Database (TFEID: Chen and Yen, 2007). All of the photos were front-view images with a standard size of 12.5 cm × 9.4 cm of six male and six female models (each with a sad and neutral expression). All of images were cropped such that only the faces (without the hairlines) of the models in front of a black background were shown. The face stimuli were gray scaled, then normalized for mean luminance and contrast across the input set using the lumMatch function in SHINE toolbox of MATLAB. The output images were equated in mean luminance (*M* = 92.9023 cd/m^2^) and contrast derived using imstats function.

### Apparatus

#### Eye-Tracking System

The EyeLink 1000 system was used to track eye movements. The system estimated gaze points by sensing pupil-center corneal reflections. Fixations in the EyeLink system were identified through calculation of saccade-pick algorithm, in which the system captured and analyzed moment-to-moment velocity and acceleration of the eye to deduce on-line velocity/acceleration threshold for marking of data points. In other words, it had an online parsing system which analyzed eye position data into meaningful events and states (e.g., saccades and blinks). The default setting for Eyelink 1000 system had one sample filter active (STD), meaning that there was a mean delay of 2.96 ms, median delay of 2.95 ms and standard deviation of 0.44 ms with 1000 Hz sampling rate (SR Research Ltd, 2014). System calibration involved automatically detecting consecutive participant gaze fixation at nine grid points. The system was further interfaced with a desktop computer, and facial expression images were shown in 32-bit color on a 1,280 × 1,024 CRT monitor. The monitor was of size 40 cm × 30 cm, had a screen refresh rate of 120 Hz and minimum display lag (<8.3 ms) (MacKenzie and Ware, 1993). The stimuli were presented using the SR Research Experiment Builder version 1.06.01.

### Procedure

The experiment was conducted individually in an eye-tracking laboratory. The study was approved by the Joint Chinese University of Hong Kong – New Territories East Cluster Clinical Research Ethics Committee (Hong Kong) and written informed consent was obtained from all participants. Upon receiving written informed consent, the participants completed the SCID and questionnaire on demographics, RRS-brooding and BDI. They were then instructed to perform the mixed antisaccade task.

For this task, participants identified their dominant eye and then sat 70 cm away from a computer screen, resting their chin on a chinrest and leaning forward onto a forehead rest to minimize any subsequent head movements during data collection. The eye fixation position of participant’s dominant eye was traced, and the system was calibrated. The participants were then told that during each trial, they were to fixate their gaze on a cross (0.4° × 0.4°) located at the center of the screen at the beginning. Based on Antoniades et al. (2013) standardized antisaccade protocol, the duration of this fixation cross was set to vary randomly across trials with a range of 1–3.5 s and a mean of 1.5 s to avoid anticipatory effects. Following on, a cue of 300 ms would appear at the center of the screen and that a facial image (*x* = 320, *y* = 512 for left side; *x* = 960, *y* = 512 for right side, 9.5° × 11.1°) would appear on either the left or right side of the screen. When the cue presented was a white oval (0.6° × 0.5°), participants were required to direct their gaze as quickly as possible to the facial image (prosaccade trial). When the cue displayed was a white cross (0.4° × 0.5°), participants were required to direct their gaze to the opposite side of the image (antisaccade trial). Participants were told to respond as quickly as they can in each trial, with experimenter pointing to the approximate correct fixation position for the prosaccade and antisaccade trials during instructions. Eye gaze data were collected at a sampling rate of 1,000 Hz, and saccade latencies of the participants’ responses on all trials were recorded. A total of 24 practice trials distributed over prosaccade, antisaccade, and mixed saccade blocks were given at the beginning of each block condition to ensure that the participants understood the instructions.

**FIGURE 1:**
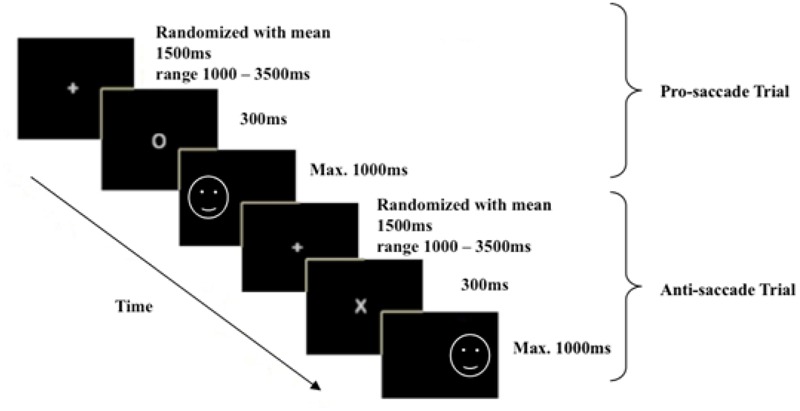
**An example of the mixed block trials used.** The white circle denotes prosaccade trials, and the white cross denotes antisaccade trials.

To minimize affective order effects, all participants completed the task in the same order: the neutral version first, followed by the sad version. For each version, the participants completed a sequence of pure prosaccade block (60 trials), pure antisaccade block (60 trials), mixed saccade block (61 trials), mixed saccade block (61 trials), pure antisaccade block (60 trials), and pure prosaccade block (60 trials) based on Antoniades et al. (2013) standardized antisaccade protocol. An example of typical trials used in the mixed saccade block was illustrated in **Figure 1**. In total, each participant completed 724 trials over 12 blocks. The distribution of trials was listed in **Table 1**. The entire test took approximately 45–50 min to complete with break times in between tasks. The participants were thanked, debriefed, and given approximately USD$25 in compensation for their time and travel expenses at the end of the experimental session.

**Table 1:** Distribution of trial types in the modified mixed antisaccade task.

Trial types
**In each version (sad face or neutral face) of the task:**
*Trials without set shift*
120 prosaccade trials (A–A)
120 antisaccade trials (B–B)
*Trials requiring a set shift*
40 inhibitory trials (20 trials of A–B–A; 20 trials of B–A–B)
40 non-inhibitory switch trials (20 trials of B–B–A; 20 trials of A–A–B)
40 unclassified trials (20 trials of B–A–B; 20 trials of A–B–B)
**Measures of executive function**
*Switch cost:*
RT to non-inhibitory switch trials – RT to repeat trials
(RT to B–B–**A**/A–A–**B** – RT to repeat A–A–**A**/B–B–**B**)
*Backward inhibition:*
RT to inhibitory trials – RT to non-inhibitory switch trials
(RT to A–B–**A**/B–A–**B** - RT to B–B–**A**/A–A–**B)**

*A represents prosaccade trials and B represents antisaccade trials.*

## Results

### Error Rate Analysis

**FIGURE 2:**
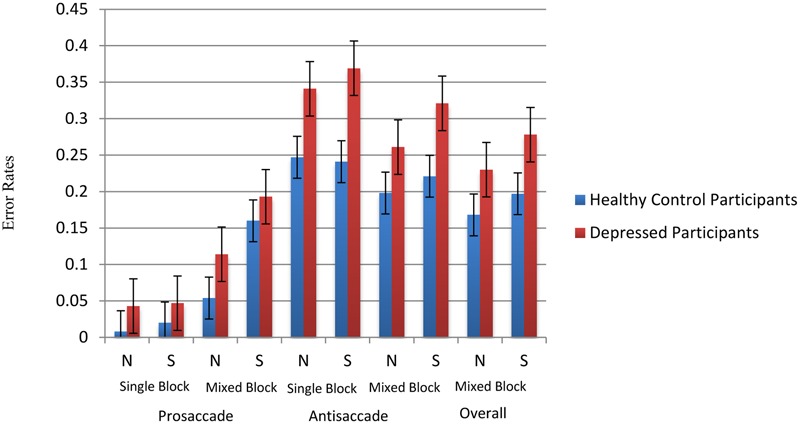
**Error rates across conditions.** N stands for neutral face version and S stands for sad face version.

The average calibration accuracy of all participants is 0.5° and standard error of calibration is 0.03°. In the present sample, the initial saccades made to the face had a mean distance of 7.88° from the fixation point. Consistent with the existing literature, saccades were defined as eye movements with velocities of over 30°/s and with amplitudes of over 3° made between onset and offset of a stimulus (Massen, 2004; De Lissnyder et al., 2011). For prosaccade trials, correct saccade was defined as the first saccade after cue onset landing on the face stimuli, which was located at the rectangular area of 10 cm × 11.7 cm located 5 cm away from the left or right edge of the screen. For antisaccade trials, correct saccade was defined by the first saccade away from the face with amplitudes of over 7° after cue onset. Trials with saccade latency less than 50 ms (i.e., anticipatory trials) were also excluded. Based on these criteria, four depressed participants (one male and three females) had an accuracy rate two standard deviations lower than the mean and were excluded from subsequent analysis. For all remaining participants, an overall accuracy rate of 82.75% was obtained. In general, depressed patients (mean = 21.4%, standard error = 2.2%) showed a higher overall error rate than healthy participants (mean = 14.1%, standard error = 1.9%) [*F*(1,32) = 6.033, *p* = 0.02]. In antisaccade trials, depressed patients (mean = 33.6%, standard error = 3.5%) made significantly more errors than healthy participants (mean = 21.9%, standard error = 3.0%) [*F*(1,32) = 6.025, *p* = 0.02]. Details of error rates across conditions were shown in **Figure 2**.

To examine error rates associated with brooding, multilevel modeling analysis (i.e., hierarchical liner modeling) was employed using the statistical package HLM (version 7) with overall accuracy rate as outcome variable. Task type (neutral faces versus sad faces) input as level-1 variable. Group, standardized RRS-brooding scores and group × standardized RRS-brooding were input as level-2 variables. There was a trend of differences observed in the main effect of group [*t*(31) = 1.766, *p* = 0.087]. All other main and interactions effects were non-significant.

### Switch Cost Analysis

**FIGURE 3:**
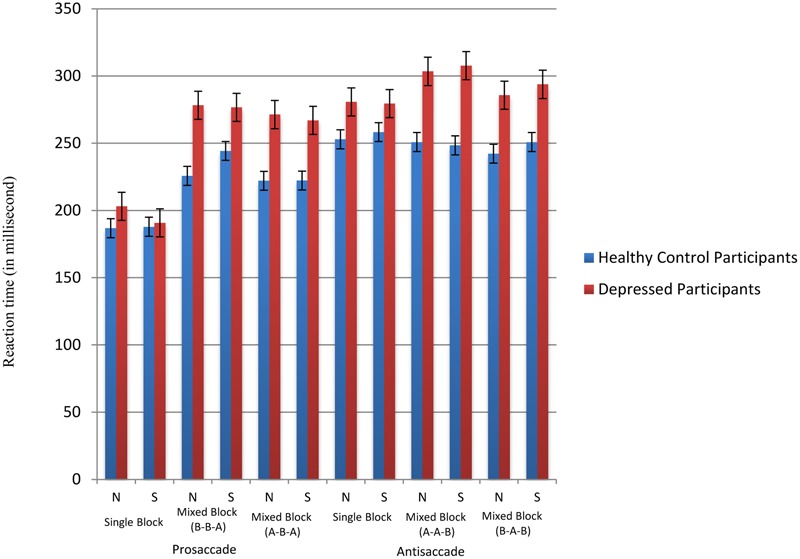
**Reaction time latencies across conditions.** N stands for neutral face version and S stands for sad face version. A represents prosaccade trials and B represents antisaccade trials.

For reaction time latencies, descriptive statistics across groups and task conditions were summarized in **Figure 3**. To facilitate analysis of switching performance, switch costs were calculated by subtracting the average saccade latencies in the pure block from those of the non-inhibitory switching trials in the mixed block (e.g., BBA-AAA or AAB-BBB). In general, depressed participants (neutral face mean = 48.88 ms, sad face mean = 57.03 ms) showed significantly greater switch costs than healthy participants (neutral face mean = 18.35 ms, sad face mean = 23.27 ms) across both neutral face [*F*(1,34) = 8.93, *p* < 0.01] and sad face conditions [*F*(1,34) = 5.60, *p* < 0.01]. Also, as seen in **Figure 3**, the prosaccade trials generally require shorter response time to perform than antisaccade trials, the switch cost involving prosaccade-following-antisaccade trials (neutral face mean = 54.45 ms, sad face mean = 69.06 ms) is generally greater in than that in antisaccade-following-prosaccade trials (neutral face mean = 8.51 ms, sad face mean = 6.42 ms) across both neutral face [*t*(34) = -3.36, *p* < 0.01] and sad face conditions [*t*(34) = -6.61, *p* < 0.01].

**FIGURE 4:**
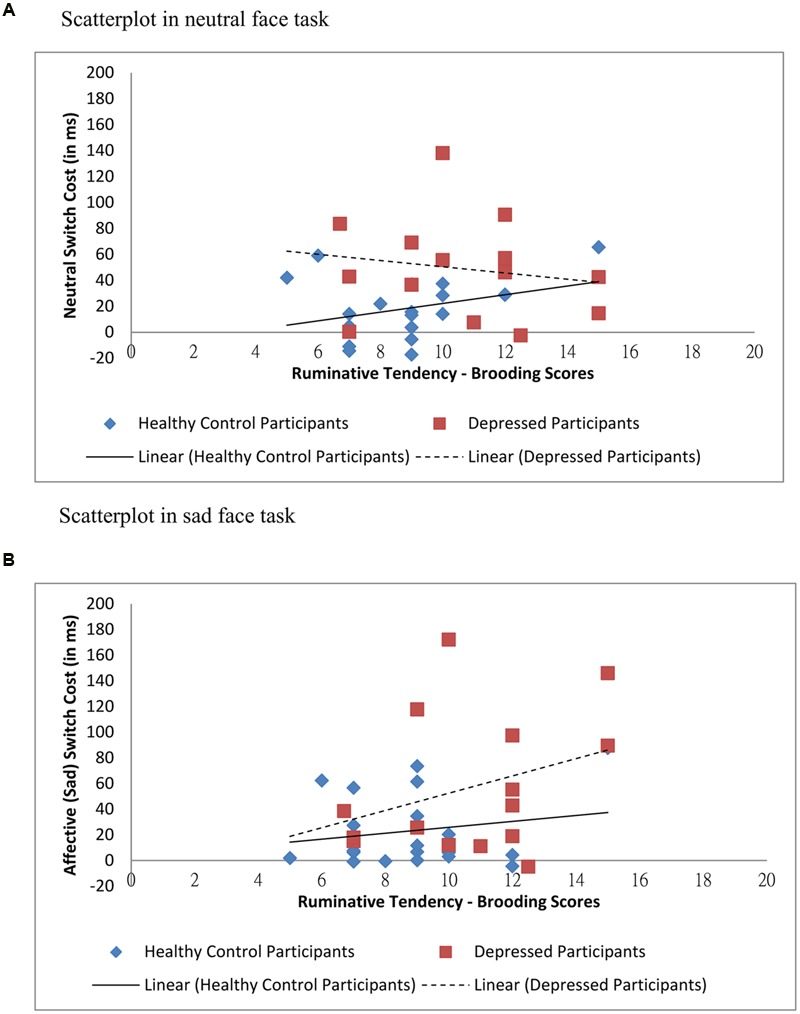
**Relations between ruminative tendency (brooding) and switch costs across groups and conditions. (A)** Scatterplot in neutral face task. **(B)** Scatterplot in sad face task.

To assess brooding associated switching efficiency across conditions, multilevel modeling analysis was again employed with standardized switch cost entered as outcome variable. Task type (neutral faces versus sad faces) was input as level-1 variable, and group, standardized RRS-brooding scores and group × standardized RRS-brooding were input as level-2 variables. Statistics showed that the main effect of group [*t*(31) = 2.307, *p* = 0.028] was significant, with depressed participants’ switch costs more than double (mean = 52.96 ms) that of the healthy controls (mean = 20.81 ms). The main effect of standardized RRS-brooding [*t*(31) = 0.924, *p* > 0.05] and the main effect of task type [*t*(31) = 0.486, *p* > 0.05] were non-significant. As hypothesized, the three-way task type × group × standardized RRS-brooding scores interaction was significant [*t*(31) = 2.014, *p* = 0.053], with switch costs in sad face condition positively correlated with brooding tendency in depressed group but not in healthy control group. The pattern of result was shown in **Figure 4**. All other two-way and three-way interactions were non-significant.

### Inhibition Cost Analysis

In antisaccade trials, depressed patients exhibited slightly longer reaction times (mean = 285.68 ms, standard error = 12.21 ms) than healthy controls (mean = 254.87 ms, standard error = 10.47 ms) across conditions [*F*(1,32) = 3.44, *p* = 0.073]. For BI analysis, inhibition costs were calculated by subtracting the average saccade latencies in the non-inhibitory switching trials from the BI trials in the mixed block (e.g., ABA-BBA or BAB-AAB). Multilevel modeling analysis was then employed with standardized inhibition cost entered as outcome variable. Task type (neutral faces versus sad faces) was input as level-1 variable, and group, standardized RRS-brooding scores and group × standardized RRS-brooding were input as level-2 variables. Statistics showed that the main effect of group [*t*(31) = -0.177, *p* > 0.05], the main effect of standardized RRS-brooding [*t*(31) = -0.507, *p* > 0.05] and the main effect of task type [*t*(31) = -0.179, *p* > 0.05] were all non-significant. All two-way and three-way interactions were also non-significant.

## Discussion and Conclusion

To our knowledge, the present study is the first to test rumination associated BI as well as switching difficulties using an affective version of the mixed antisaccade task. Although null findings were observed for association between brooding tendency and BI, our results found that brooding tendency was positively associated with switching inefficiency particularly when depressed patients were processing the sad face task. These results contrasted with De Lissnyder et al.’s (2011) null findings when neutral stimuli were applied, and echoed previous studies adopting manual responses to suggest that stimulus affectivity and mood to be critical factors that mitigate set shifting difficulties associated with trait rumination (Whitmer and Banich, 2007; De Lissnyder et al., 2010).

The switch cost pattern observed adds to a growing body of evidence to suggest that set shifting difficulties associated with rumination may be specifically modulated by negative mood and cognition. Whitmer and Gotlib (2013) proposed an attention scope theory in explaining these observations, and argue that affective experiences may influence cognitive processing by altering the scope of attention focus. Negative affective experiences may narrow attention focuses and thereby limit information activation in working memory, leading to a slowing of conceptual change. For people who are inherently characterized by a constricted attention scope (i.e., high ruminators), depressive mood states further trigger restrictions on their control functions such that substantially more cognitive resources are needed to disengage from one mental representation. Hence, high ruminators would experience greater difficulty to flexibly switch to new mental rules/sets. Yet, a major concern relates to that the present study was cross-sectional in nature, and our results cannot infer causation. In other words, we are not sure whether trait rumination leads to switching difficulties, whether individuals with switching deficits are prone to ruminate, or whether a third latent variable affects both factors. Further longitudinal studies are required to delineate whether impairments in task switching ability can be seen as antecedents, consequences, or epiphenomena of rumination. As such, the modulating effect of the depressive status observed needs to be cautiously interpreted, as there is an alternative possible explanation that individuals with higher brooding tendency may engage in more ruminative thinking during a depressed state (especially when looking at sad facial images). In other words, the driving factor could be related to individual’s state rumination rather than trait rumination, and reduced task switching ability may be a consequence of heightened rumination state, as opposed to a deficit associated with trait rumination activated under depressed mood state.

In terms of accuracy rate analysis, our results indicated that depressed participants showed higher error rates than healthy participants across conditions. In antisaccade trials in particular, depressed participants committed at least 50% more errors than healthy participants. The results were consistent with Derakshan et al. (2009) and Carvalho et al. (2014) in which dysphoric individuals showed greater difficulty making response inhibition in antisaccade conditions. In similar vein, latency analysis of antisaccade trials suggested that depressed patients tended to exhibit slightly longer reaction times than healthy participants. However, no significant interaction with brooding tendency or task types was observed. With regard to BI function (i.e., control of reactivating a previously abandoned mental rule or task goal), no association was found with brooding trait or depressed states in the present analysis. This was somewhat inconsistent with previous literature (Whitmer and Banich, 2007; De Lissnyder et al., 2010; Whitmer and Gotlib, 2012) when manual motor response was adopted. However it should be noted that in the present study, only prosaccade and antisaccade task rules were administered and the deductions of BI measure is not as ideal as in previous studies in which a third control condition can be used for comparison in deducing a purer BI measure (e.g., ABA-CBA). In addition, emotional responses toward only sad and neutral faces were examined, as opposed to previous studies adopting either neutral stimuli (Whitmer and Banich, 2007; Whitmer and Gotlib, 2012), or happy versus angry faces (De Lissnyder et al., 2010). It remained unknown whether other emotional faces (e.g., angry faces, happy faces) would elicit the same pattern observed among depressed ruminators, as affective valence has been observed to be a key factor to influencing inhibition abilities in antisaccade tasks (Derakshan et al., 2009; García-Blanco et al., 2013). Furthermore, if emotional value of the face is important in peripheral visual perception, then the facial target size might also be a crucial factor to explore in future research as the facial cues for sad versus happy or angry emotions could be different.

The present study has some limitations. First, while we targeted non-inhibitory switching performance and minimized inhibition interference by focusing on specific non-inhibitory switch trials (e.g., AAB), we could not fully exclude response inhibition effects. This is because antisaccade trials require participants to inhibit eye movements and to generate a volitional saccade to the opposite side of the cue, and as such, the switch cost deduced closely represents but does not stand alone as a pure index of switching efficiency. In the present study, depressed patients showed higher error rates and longer latency in antisaccade trials as compared to healthy participants, indicating some deficits in response inhibition. However, no association with brooding trait was found and future research could further explore if response inhibition effects and non-inhibitory switching effects could be delineated in other experimental task designs. Second, the order of blocks was fixed in the present design, that is, neutral face version was presented first before sad face version for all participants. Future studies could counterbalance the order to investigate the block effect, as well as extending the analysis to examine training or fatigue effects over block of trials (so far, there is null effect on blocks in the present design). Third, we recruited a depressed sample in the present study, and by nature many of these individuals were high ruminators and were medicated. Moreover, there was a possibility that the depressed patients and healthy controls may differ in other regards (e.g., socio-economic status, life stress) than just depressive states. Future studies may be required to examine whether stimulus affectivity and mood state effects may be replicated among non-clinical samples that are free of medication use and are homogenous in terms of background profiles.

Albeit the above limitations, the design has its strengths as incorporation of facial images simulates everyday life context in a practical way. It mimics rumination phenomenology in daily situations where individuals with depression susceptibility receive visual cues from others’ facial expressions (possibly triggering all sorts of mood-congruent memory retrieval and/or negative analytical interpretations in interpersonal and self-referential contexts), making it hard to disengage from these visual facial cues to focus on other cognitive tasks. In summary, the present study showed that the modified mixed antisaccade task serves as a useful and valid mean of examining executive control performance associated with rumination, and future prospective studies could consider adopting it to further verify trait versus state rumination effects.

## Ethics Statement

This study was carried out in accordance with the recommendations of The Joint Chinese University of Hong Kong – New Territories East Cluster Clinical Research Ethics Committee (Hong Kong) with written informed consent from all subjects. All subjects gave written informed consent in accordance with the Declaration of Helsinki. The protocol was approved by the The Joint Chinese University of Hong Kong – New Territories East Cluster Clinical Research Ethics Committee (Hong Kong).

## Author Contributions

BL contributed to study design, data collection and processing, statistical analysis, interpretation, and paper writing. JL contributed to data collection, clinical supervision, and paper review.

## Conflict of Interest Statement

The authors declare that the research was conducted in the absence of any commercial or financial relationships that could be construed as a potential conflict of interest.

## References

[B1] Antoniades C. Ettinger U. Gaymard B. Gilchrist I. Kristjansson A. Kennard C. (2013). An internationally standardised antisaccade protocol. *Vision Res.* 84 1–5. 10.1016/j.visres.2013.02.007 23474300

[B3] Bagby R. M. Rector N. A. Bacchiochi J. R. McBride C. (2004). The stability of the response styles questionnaire rumination scale in a sample of patients with major depression. *Cogn. Ther. Res.* 28 527–538. 10.1023/B:COTR.0000045562.17228.29

[B4] Beck A. T. Steer R. A. Brown G. K. (1996). *Manual for the Beck Depression Inventory-II*. San Antonio, TX: Psychological Corporation.

[B5] Berman M. G. Nee D. E. Casement M. Kim H. S. Deldin P. Kross E. (2011). Neural and behavioral effects of interference resolution in depression and rumination. *Cogn. Affect. Behav. Neurosci.* 11 85–96. 10.3758/s13415-010-0014-x 21264648 PMC4006074

[B6] Bernblum R. Mor N. (2010). Rumination and emotion-related biases in refreshing information. *Emotion* 10 423–432. 10.1037/a0018427 20515230

[B7] Carvalho N. Noiret N. Vandel P. Monnin J. Chopard G. Laurent E. (2014). Saccadic eye movements in depressed elderly patients. *PLoS ONE* 9:e105355 10.1371/journal.pone.0105355 PMC4133355 25122508

[B8] Chen L. F. Yen Y. S. (2007). *Taiwanese Facial Expression Image Database*. Taipei: National Yang-Ming University.

[B9] Davis R. N. Nolen-Hoeksema S. (2000). Cognitive inflexibility among ruminators and nonruminators. *Cogn. Ther. Res.* 24 699–711. 10.1023/A:1005591412406

[B10] De Lissnyder E. Derakshan N. De Raedt R. Koster E. H. (2011). Depressive symptoms and cognitive control in a mixed antisaccade task: specific effects of depressive rumination. *Cogn. Emot.* 25 886–897. 10.1080/02699931.2010.514711 21824026

[B11] De Lissnyder E. Koster E. H. De Raedt R. (2012). Emotional interference in working memory is related to rumination. *Cogn. Ther. Res.* 36 348–357. 10.1007/s10608-011-9352-4

[B12] De Lissnyder E. Koster E. H. Derakshan N. De Raedt R. (2010). The association between depressive symptoms and executive control impairments in response to emotional and non-emotional information. *Cogn. Emot.* 24 264–280. 10.1080/02699930903378354

[B13] Derakshan N. Salt M. Koster E. H. (2009). Attentional control in dysphoria: an investigation using the antisaccade task. *Biol. Psychol.* 80 251–255. 10.1016/j.biopsycho.2008.09.005 18950676

[B14] First M. B. Spitzer R. L. Gibbon M. Williams J. B. W. (2002). *Structured Clinical Interview for DSM-IV-TR Axis I Disorders. Research Version, Non-patient Edition (SCID-I/NP)*. New York, NY: New York State Psychiatric Institute.

[B15] Foland-Ross L. C. Hamilton J. P. Joormann J. Berman M. G. Jonides J. Gotlib I. H. (2013). The neural basis of difficulties disengaging from negative irrelevant material in major depression. *Psychol. Sci.* 24 334–344. 10.1177/0956797612457380 23334445 PMC4004633

[B16] García-Blanco A. Perea M. Salmerón L. (2013). Attention orienting and inhibitory control across the different mood states in bipolar disorder: an emotional antisaccade task. *Biol. Psychol.* 94 556–561. 10.1016/j.biopsycho.2013.10.005 24161800

[B17] Joormann J. (2006). Differential effects of rumination and dysphoria on the inhibition of irrelevant emotional material: evidence from a negative priming task. *Cogn. Ther. Res.* 30 149–160. 10.1007/s10608-006-9035-8

[B18] Joormann J. Gotlib I. H. (2008). Updating the contents of working memory in depression: interference from irrelevant negative material. *J. Abnorm. Psychol.* 117 182–192. 10.1037/0021-843X.117.1.182 18266496

[B19] Joormann J. Levens S. M. Gotlib I. H. (2011). Sticky thoughts: depression and rumination are associated with difficulties manipulating emotional material in working memory. *Psychol. Sci.* 22 979–983. 10.1177/0956797611415539 21742932 PMC11862919

[B20] Just N. Alloy L. B. (1997). The response styles theory of depression: tests and an extension of the theory. *J. Abnorm. Psychol.* 106 221–229. 10.1037//0021-843X.106.2.221 9131842

[B21] Koster E. H. De Lissnyder E. De Raedt R. (2013). Rumination is characterized by valence-specific impairments in switching of attention. *Acta Psychol.* 144 563–570. 10.1016/j.actpsy.2013.09.008 24140824

[B22] Koster E. H. De Lissnyder E. Derakshan N. De Raedt R. (2011). Understanding depressive rumination from a cognitive science perspective: the impaired disengagement hypothesis. *Clin. Psychol. Rev.* 31 138–145. 10.1016/j.cpr.2010.08.005 20817334

[B23] Krompinger J. W. Simons R. F. (2011). Cognitive inefficiency in depressive undergraduates: stroop processing and ERPs. *Biol. Psychol.* 86 239–246. 10.1016/j.biopsycho.2010.12.004 21185350

[B24] Lau M. A. Christensen B. K. Hawley L. L. Gemar M. S. Segal Z. V. (2007). Inhibitory deficits for negative information in persons with major depressive disorder. *Psychol. Med.* 37 1249–1259. 10.1017/S0033291707000530 17451630

[B25] Lo B. C. Y. Lau S. Cheung S. H. Allen N. B. (2012). The impact of rumination on internal attention switching. *Cogn. Emot.* 26 209–223. 10.1080/02699931.2011.574997 21614702

[B26] MacKenzie I. S. Ware C. (1993). “Lag as a determinant of human performance in interactive systems,” in *Proceedings of the ACM Conference on Human Factors in Computing Systems INTERCHI* Vol. 93 (New York, NY: ACM New York) 488–493. 10.1145/169059.169431

[B27] Massen C. (2004). Parallel programming of exogenous and endogenous components in the antisaccade task. *Q. J. Exp. Psychol. A* 57 475–498. 10.1080/02724980343000341 15204137

[B28] Nolen-Hoeksema S. Morrow J. (1991). A prospective study of depression and posttraumatic stress symptoms after a natural disaster: the 1989 Loma Prieta Earthquake. *J. Pers. Soc. Psychol.* 61 115–121. 10.1037/0022-3514.61.1.115 1890582

[B29] Piguet C. Cojan Y. Sterpenich V. Desseilles M. Bertschy G. Vuilleumier P. (2016). Alterations in neural systems mediating cognitive flexibility and inhibition in mood disorders. *Hum. Brain Mapp.* 37 1335–1348. 10.1002/hbm.23104 26787138 PMC6867498

[B30] SR Research Ltd (2014). *EyeLink 1000 Technical Specifications.* Available at: http://www.sr-research.com/pdf/techspec.pdf

[B31] Whitmer A. J. Banich M. T. (2007). Inhibition versus switching deficits in different forms of rumination. *Psychol. Sci.* 18 546–553. 10.1111/j.1467-9280.2007.01936.x 17576269

[B32] Whitmer A. J. Gotlib I. H. (2012). Switching and backward inhibition in major depressive disorder: the role of rumination. *J. Abnorm. Psychol.* 121 570–578. 10.1037/a0027474 22468767 PMC11877650

[B33] Whitmer A. J. Gotlib I. H. (2013). An attentional scope model of rumination. *Psychol. Bull.* 139 1036–1061. 10.1037/a0030923 23244316 PMC3773498

